# A WRKY Transcription Factor Recruits the SYG1-Like Protein SHB1 to Activate Gene Expression and Seed Cavity Enlargement

**DOI:** 10.1371/journal.pgen.1003347

**Published:** 2013-03-07

**Authors:** Xiaojun Kang, Wei Li, Yun Zhou, Min Ni

**Affiliations:** Department of Plant Biology, University of Minnesota at Twin Cities, Saint Paul, Minnesota, United States of America; Keck Graduate Institute of Applied Life Sciences, United States of America

## Abstract

Seed development in Arabidopsis and in many dicots involves an early proliferation of the endosperm to form a large embryo sac or seed cavity close to the size of the mature seed, followed by a second phase during which the embryo grows and replaces the endosperm. SHORT HYPOCOTYL UNDER BLUE1 (SHB1) is a member of the SYG1 protein family in fungi, *Caenorhabditis elegans*, flies, and mammals. SHB1 gain-of-function enhances endosperm proliferation, increases seed size, and up-regulates the expression of the WRKY transcription factor gene *MINISEED3* (*MINI3*) and the LRR receptor kinase gene *HAIKU2* (*IKU2*). Mutations in either *IKU2* or *MINI3* retard endosperm proliferation and reduce seed size. However, the molecular mechanisms underlying the establishment of the seed cavity and hence the seed size remain largely unknown. Here, we show that the expression of *MINI3* and *IKU2* is repressed before fertilization and after 4 days after pollination (DAP), but is activated by SHB1 from 2 to 4 DAP prior to the formation of the seed cavity. SHB1 associates with their promoters but without a recognizable DNA binding motif, and this association is abolished in *mini3* mutant. MINI3 binds to W-boxes in, and recruits SHB1 to, its own and *IKU2* promoters. Interestingly, SHB1, but not MINI3, activates transcription of *pMINI3*::*GUS* or *pIKU2*::*GUS*. We reveal a critical developmental switch through the activation of *MINI3* expression by SHB1. The recruitment of SHB1 by MINI3 to its own and *IKU2* promoters represents a novel two-step amplification to counter the low expression level of *IKU2*, which is a trigger for endosperm proliferation and seed cavity enlargement.

## Author Summary

Seed development in many dicots is characterized by a rapid proliferation of the endosperm and growth of integument to form a large embryo sac or seed cavity. In Arabidopsis, the seed cavity is generated at the globular stage or 4 days after pollination. The subsequent growth of the embryo replaces the endosperm during the second phase. Therefore, the volume of the initial seed cavity correlates closely with the final seed size. In *shb1-D*, an even larger seed cavity is created at 4 DAP due to an up-regulated expression of *MINI3* and *IKU2* by SHB1. We report that the expression of *MINI3* and *IKU2* coincides with the formation of the seed cavity. SHB1 is anchored to these promoters by MINI3 to activate their expression in a W-box-dependent manner. Spatiotemporal regulation of gene expression is a crucial mechanism that controls embryo development in many organisms. This interaction of SHB1 with MINI3 should impact studies of their homologs in many other organisms, including humans. Seed development in major seed crops, such as soybean and canola, follows a very similar path to that of *Arabidopsis*. Our results should lead to an increase in agricultural yields and concomitant increases in the proteins and oil content per seed.

## Introduction

In angiosperms, double fertilization leads to the formation of a diploid embryo and a triploid endosperm, and the endosperm arises from the central cell that contains two identical haploid genomes. In many dicots, such as *Arabidopsis*, seed development follows two distinct phases and the embryo grows to full size and replaces most of the endosperm at maturity [1], [2]. During the first phase, the syncytial phase, a rapid growth and proliferation of the endosperm occurs, which generates a large multinucleate cell and results in a larger embryo sac or seed cavity by 4 DAP [3]–[5]. This syncytium is then partitioned into individual cells by a specific type of cytokinesis called cellularization [6]. During the second phase, embryo growth takes place at the expense of the endosperm. Upon maturity, the seed contains only a single layer of endosperm cells in *Arabidopsis*, and the maternal integument ultimately becomes the seed coat [6], [7]. The seed coat and endosperm growth in *Arabidopsis* precedes embryo growth, and the seed reaches almost its final size before the embryo enlarges. Both maternal and non-maternal factors are involved in seed size regulation [8].

In *Arabidopsis*, increased dosage of the paternal genome in the endosperm increases seed size whereas increased dosage of the maternal genome causes the opposite effect [9]. Defects in endosperm development include delayed cellularization of the peripheral endosperm and hypertrophy of the chalazal endosperm and associated nodules [7]. Mutations in *DNA METHYLTRANSFERASE1* (*MET1*) and *DECREASE IN DNA METHYLATION1* (*DDM1*) reduce DNA methylation dramatically and cause parent-of-origin effects on F1 seed size [9]. Pollination of *met1-6* or *ddm1-2* pistils with wild-type pollens produced large F1 seeds, and reciprocal crosses generated small F1 seeds. Mutations in the transcription factor *APETALA2* (*AP2*) increased seed size due to an increase in both embryo cell number and cell size [10], [11]. The seed trait is passed via the maternal sporophyte and endosperm genomes.

In contrast, mutations in either *IKU* (*HAIKU*) or *MINI3* (*MINISEED3*) reduce seed size, and the mutant seed phenotypes depend on the genotype of the embryo and the endosperm but not on the genotype of the maternal ovule [12]. In Arabidopsis, a larger embryo sac or seed cavity, coordinated by the growth of the maternal integument and the endosperm, is created at 4 DAP [1], [2]. TRANSPARENT TESTA GLABRA2 (TTG2), METHYLTRANSFERASE1 (MET1), and MEGAINTEGUMENTA/AUXIN RESPONSE FACTOR 2 (MNT/ARF2) regulate integument growth [8], [13], [14] whereas MINI3, IKU2, IKU1, and SHB1 control endosperm proliferation [12], [15], [16]. Mutations in either *MINISEED3* (*MINI3*) or *HAIKU2* (*IKU2*) cause premature cellularization of the endosperm, reduced growth of the endosperm including the chalazal endosperm, and reduced proliferation of the embryo after the early torpedo stage [6], [12]. *SHB1* was initially isolated from a gain-of-function overexpression mutant, *short hypocotyl under blue 1 Dominant* (*shb1-D*), based on its long hypocotyl phenotype under red, far-red, and blue light [17]. *shb1-D* significantly increased seed mass and the total seed yield compared with Ws wild type [15]. In *shb1-D*, an even larger seed cavity is created at 4 DAP along with an enlarged chalazal endosperm and a delay in endosperm cellularization. AGL62 also regulates endosperm cellularization and the endosperm cellularizes prematurely in *agl62* seeds [18].

*MINI3* or the *WRKY10* gene encodes a WRKY family transcription factor [12]. *MINI3* is expressed in the endosperm and the embryo from 12 to 96 hr after fertilization but not in the late-heart embryo at 110 hr after fertilization or the unfertilized ovule [12]. *IKU2* encodes a leucine-rich repeat (LRR) receptor kinase. *IKU2* expression was visible in the endosperm at 12 and 48 hr post-fertilization but not in the embryo or elsewhere in the plant [12]. SHB1 contains an N-terminal SPX domain and a C-terminal EXS domain and is homologous to the SYG1 protein family members of fungi, *C. elegans*, flies, and mammals [17], [19]. Unlike the location of many SYG1-like proteins to the membrane, SHB1 is localized to the nucleus in Arabidopsis. IKU1 is a nuclear protein with an N-terminal VQ motif [17]. *IKU1* shows a similar expression pattern to *MINI3* and *IKU2* in the early developing endosperm before cellularization as *MINI3* and *IKU2* but GFP signals in *pIKU1*:*GFP*:*IKU1* plants were also detected in the integument or seed coats [16].

The expression of *IKU2* is reduced in *mini3*, *iku1*, and *shb1*, and the expression of *MINI3* is reduced in *iku1* and *shb1* [12], [15]. In this study, we observed that the expression of *MINI3* and *IKU2* is regulated stringently before fertilization and after 4 DAP, and it is activated by SHB1 at 2 and 3 DAP during endosperm proliferation. SHB1 associates with the promoters of *MINI3* and *IKU2*, and this association requires MINI3. MINI3 binds to its target W-boxes in these two promoters and interacts with the N-terminus of SHB1. Furthermore, SHB1, but not MINI3, trans-activates the expression from either a *MINI3* or an *IKU2* promoter-GUS construct.

## Results

### SHB1 regulates the spatio-temporal expression of *MINI3* and *IKU2*

**Figure 1:**
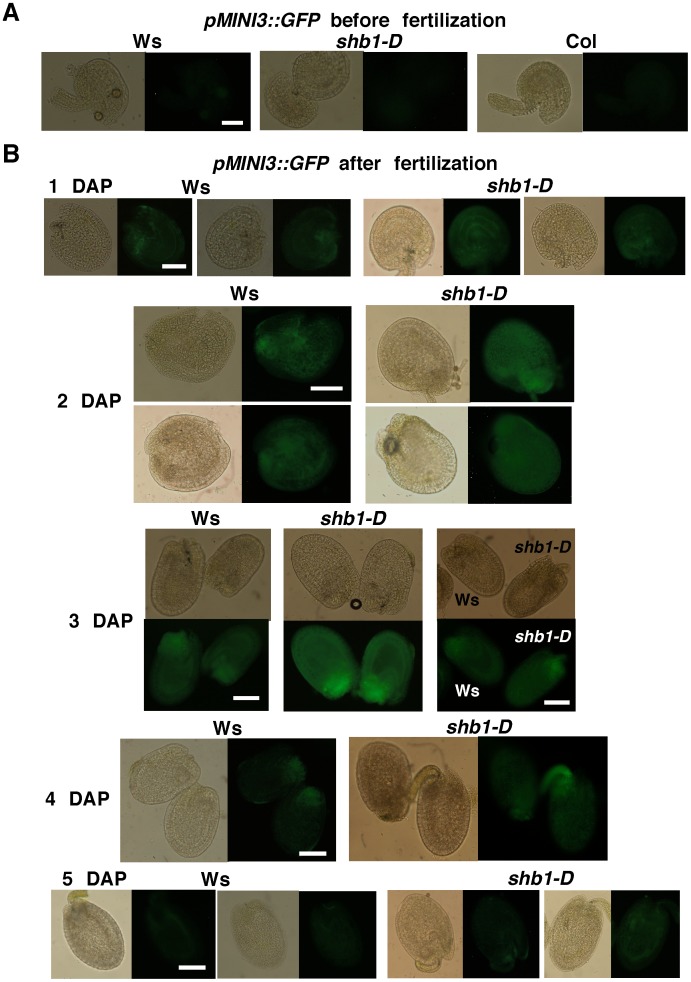
SHB1 regulates the expression of *pMINI3::GFP*. Expression of *pMINI3::GFP* in Ws wild type and *shb1-D* seeds before fertilization (A) and from 1 to 5 DAP (B). Images on the left (1 to 2 and 4 to 5 DAP) or at the top (3 DAP) are of bright field, and images on the right (1 to 2 and 4 to 5 DAP) or at the bottom (3 DAP) are of GFP fluorescence. Scale bars, 100 µm.

**Figure 2:**
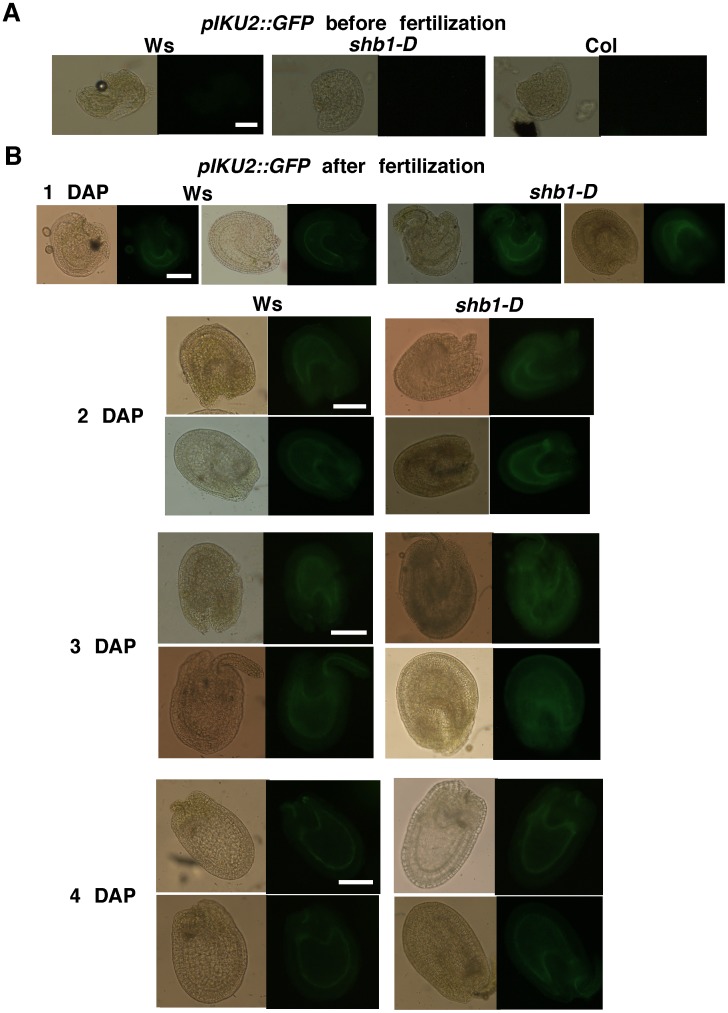
SHB1 regulates the expression of *pIKU2::GFP*. The expression of *pIKU2::GFP* in Ws wild type and *shb1-D* seeds before fertilization (A) and from 1 to 5 DAP (B). Images on the left are of bright field, and images on the right are of GFP fluorescence. Scale bars, 100 µm.

The expression of *MINI3* and *IKU2* peaks during a narrow window of up to 4 DAP, coincides with the formation of a large seed cavity, and is regulated by SHB1 [12], [15]. GUS is expressed in *MINI3*::*GUS* transgenic plants at 12 to 96 hr after fertilization in the globular and early-heart embryo and endosperm but not in the late-heart embryo at 110 hr post-fertilization [12]. GUS activity in *IKU2*::*GUS* plants was visible in endosperm at 12 and 48 hr post-fertilization and before cellularization but not in the embryo [12]. The expression of *SHB1* has been shown to overlap with *MINI3* and *IKU2* in the endosperm [15]. To refine the dynamics of *MINI3* and *IKU2* expression after pollination, we generated four independent *pMINI3::GFP* or *pIKU2*::GFP transgenic plants with a single t-DNA insertion in Ws and Col, respectively. To learn when and where SHB1 activates the expression of *MINI3* and *IKU2*, we crossed these transgenes into *shb1-D*, *shb1*, or *mini3* background (Figure 1 and Figure 2).

We examined the expression of GFP in several seeds from the same silique for each genotype and examined the expression levels in several flowers of individual plants. When recording and processing fluorescent images, the illumination was adjusted and the gain used was similar for all. GFP expression was similar within individual plants, and two representative seeds from the same silique are shown, to indicate the consensus pattern of GFP expression, in Figures 1 and 2. In either Ws or Col, *pMINI3*::*GFP* signals were not detectable in the ovules before fertilization and was weak at 1 DAP (Figures 1 and Figure S1). Activity of the *MINI3* promoter increased at 2 DAP, reached a maximum at 3 DAP, declined at 4 DAP, and was barely detectable or at background level by 5 DAP (Figure 1B and Figure S1). Activity of the *MINI3* promoter was observed in the embryo, the peripheral endosperm, and the chalazal endosperm (Figure 1B and Figure S1). In *shb1-D*, GFP signals were enhanced slightly at 1 DAP, enhanced robustly at 2 and 3 DAP, and remained enhanced at 4 and 5 DAP (Figure 1B). In contrast, activity of the *MINI3* promoter was reduced in *shb1* seeds compared with Col seeds, particularly at 2 and 3 DAP (Figure S1). Activity of *pMINI3::GFP* was not affected significantly in *mini3* (Figure S1), which is consistent with a previous report showing that MINI3 may possess an auto-regulatory function to repress its own expression [12].

Activity of *pIKU2::GFP* was not detectable in ovules before fertilization, and was weak at 1 DAP, stronger at 2 DAP, strong at 3 DAP, weak again at 4 DAP, and barely detectable at 5 DAP in Ws plants (Figure 2). GFP signals were primarily observed in the peripheral endosperm and the chalazal endosperm (Figure 2). Based on 4 independent transgenic lines for each construct and each ecotype (Figure 1 and Figure 2), the signals generated by *pIKU2::GFP* were in general much weaker than those of *pMINI3::GFP* plants. Stronger GFP activity was observed in *shb1*-*D* seeds compared with Ws seeds, particularly at 2 and 3 DAP (Figure 2A). Due to the weaker activity of the *IKU2* promoter, we were unable to detect a consistent difference in GFP signals in *shb1* or *mini3* seeds compared with Col seeds. Importantly, SHB1 activates the expression of *MINI3* and *IKU2* within a very narrow window at approximately 2 or 3 DAP and prior to the enlargement of the seed cavity.

### MINI3 ChIPs to *MINI3* and *IKU2* promoters

**Figure 3:**
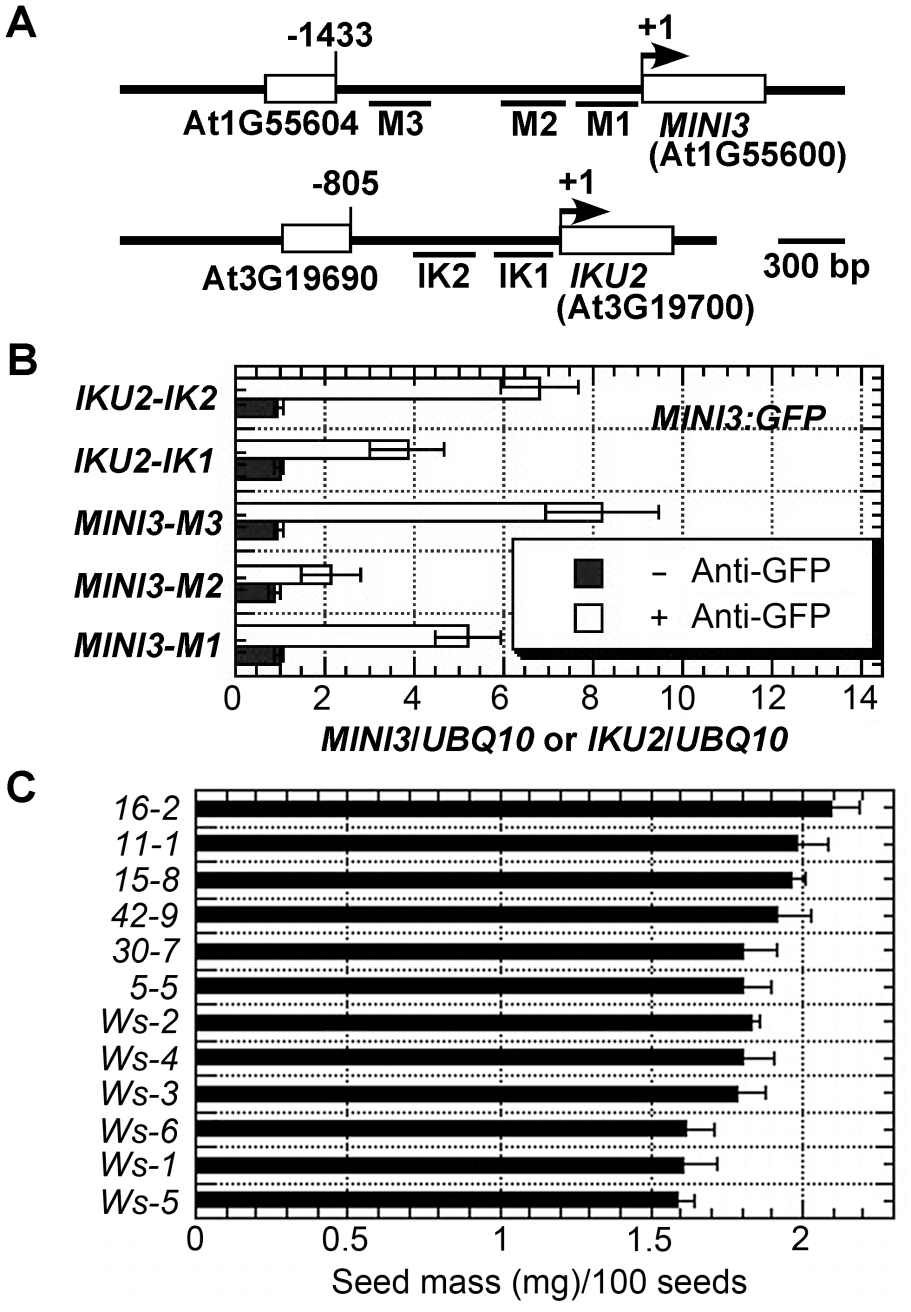
MINI3 ChIPs to the promoters of *MINI3* and *IKU2*. (A) Schematic diagram of the *MINI3* and *IKU2* loci and five amplicons (M1, M2, M3, IK1, and IK2) used for the ChIP-qPCR analysis. Rectangles represent genes and numbers indicate genomic nucleotide sequence coordination. Arrowheads indicate transcription start sites. (B) ChIP-qPCR analysis of the amplicons in the *MINI3* and *IKU2* promoters using anti-GFP monoclonal antibody in samples from 35S:MINI3:GFP transgenic plants. BSA was used as a mock control, and the fold enrichment of the specific chromatin fragments was normalized to the *UBQ10* amplicon. Means were calculated from three biological samples, each of which was calculated from three technical replicates. The calculated standard errors comprise the technical error and biological error. (C) Seed masses were examined for six independently propagated Ws lines and six independent 35::MINI3:GFP transgenic lines (5-5 to 42-9) and expressed as mg/100 seeds.

SHB1 regulates the expression of *MINI3* and *IKU2* and associates with their promoters in vivo [15]. To examine whether MINI3 is the transcription factor that anchors SHB1 to these promoters, we performed a chromatin immunoprecipitation quantitative PCR (ChIP-qPCR) assay in transgenic plants expressing *MIN3:GFP* under the control of a 35S promoter. Figure 3A shows the various amplicons in the *MINI3* and *IKU2* promoters that were used for the ChIP analysis: M1 from +6 to −348, M2 from −398 to −619, M3 from −985 to −1214, IK1 from −4 to −297, and IK2 from −278 to −566. Interestingly, MINI3 also associated with the *MINI3* promoter because both amplicon M1 and M3 were highly enriched whereas amplicon M2 in the *MINI3* promoter was not enriched significantly (Figure 3B). Amplicon 1 in the *IKU2* promoter was moderately enriched and amplicon 2 was highly enriched (Figure 3B). To determine whether the level of GFP-tagged MINI3 produced in the endosperm of 35S::MINI3:GFP transgenic plants is sufficient for ChIP analysis, we examined the seed mass of six independent 35S::MINI3:GFP transgenic lines (Figure 3C). Compared with six independently propogated Ws lines, the six 35S::MINI3:GFP transgenic lines produced relatively larger seeds.

### The association of SHB1 with *MINI3* and *IKU2* promoters requires MINI3

**Figure 4:**
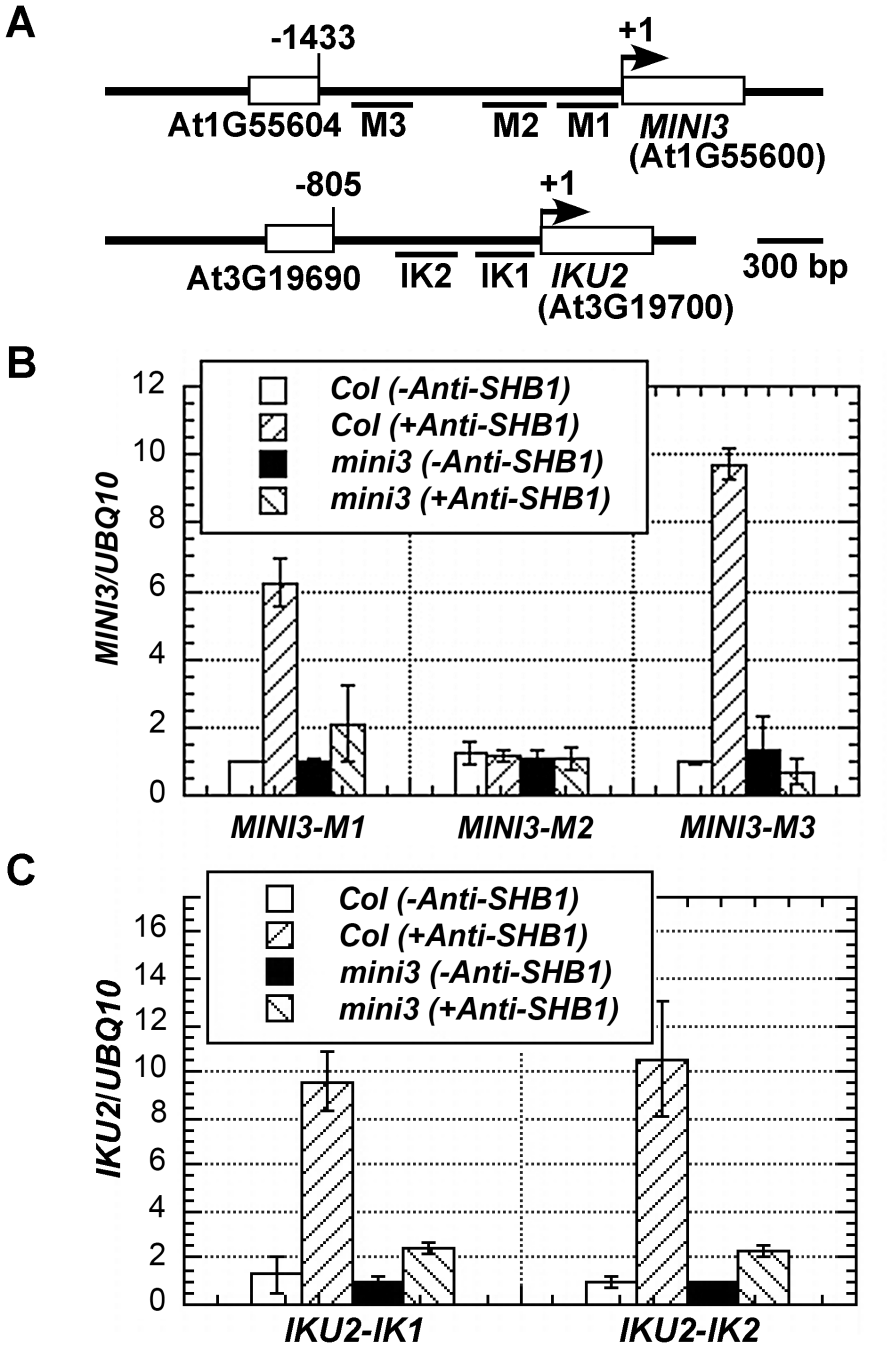
Association of SHB1 with *MINI3* and *IKU2* promoters requires MINI3. (A) A schematic diagram of the *MINI3* and *IKU2* loci and the five amplicons (M1, M2, M3, IK1, and IK2) used for the ChIP-qPCR analysis. Rectangles represent genes and numbers indicate genomic nucleotide sequence coordination. Arrowheads indicate transcription start sites. ChIP-qPCR analysis of the amplicons from the promoters of *MINI3* (B) and *IKU2* (C) using an anti-SHB1 antibody in wild type or *mini3* mutant samples. Preimmune serum was used as a mock control, and the fold enrichment of the specific chromatin fragments was normalized to the *UBQ10* amplicon. Means were calculated from three biological samples, each of was calculated from three technical replicates. The calculated standard errors comprise the technical error and biological error.

A similar pattern of fragment enrichment in the *MINI3* and *IKU2* promoters was also observed in the SHB1 ChIP assay [15]. SHB1 does not contain a recognizable DNA binding motif, and MINI3 is a WRKY transcription factor [12], [15]. MINI3 may mediate the in vivo association of SHB1 to the *MINI3* and *IKU2* promoters. We performed a ChIP analysis of SHB1 over the promoters of *MINI3* and *IKU2* in wild type and *mini3* mutant samples (Figure 4B and 4C). SHB1 associated similarly with the promoters of both *MINI3* and *IKU2* in wild-type MINI3 siliques but failed to associate with either the *MINI3* or the *IKU2* promoter when MINI3 is mutated (Figure 4B and 4C) [15]. SHB1 is most likely recruited to either the *MINI3* or the *IKU2* promoter by MINI3.

### MINI3 binds W-boxes in the *MINI3* or *IKU2* promoters

**Figure 5:**
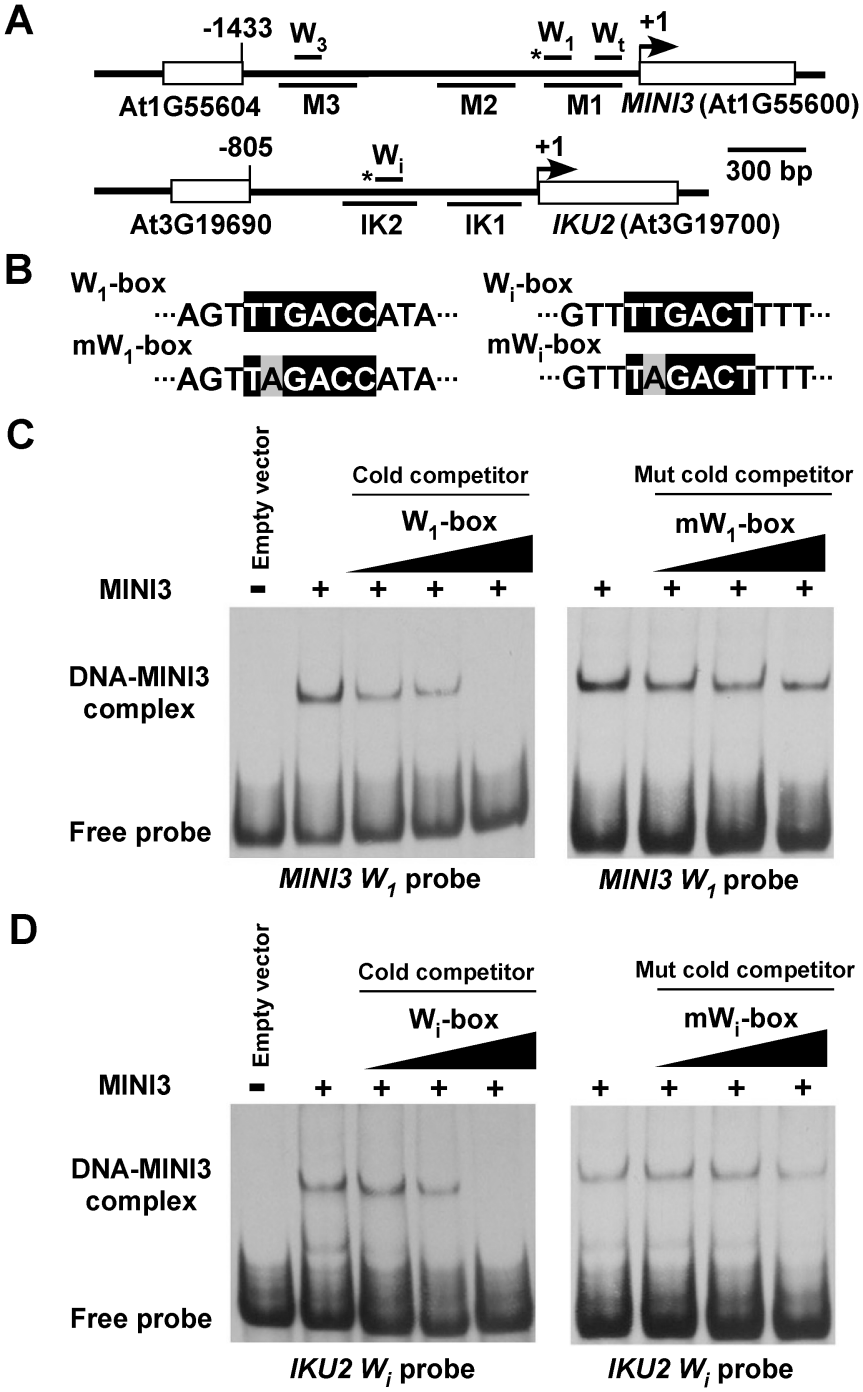
MINI3 binds to W-boxes in the *MINI3* and *IKU2* promoters. (A) A schematic diagram of the *MINI3* and *IKU2* loci, the five amplicons (M1, M2, M3, IK1, and IK2) used for the ChIP-qPCR analysis, and the position of the W-boxes in the *MINI3* and *IKU2* promoters. Rectangles represent genes and numbers indicate genomic nucleotide sequence coordination. Arrowheads indicate transcription start sites, and the asterisk indicates W-boxes recognized by MINI3. (B) The nucleotide sequences of the W_1_-box, mutated W_1_-box (mW_1_-box), W_i_-box, and the mutated W_i_-box (mW_i_-box). Core sequences are shaded in black, and mutated nucleotides are shaded in gray. EMSA analysis of the binding of MINI3 to W_1_-box in the *MINI3* promoter (C) or W_i_-box in the *IKU2* promoter (D). Cold wild type or mutated W_1_-box and W_i_-box competitors were used at a molar excess of 5X, 10X, or 50X.

*MINI3* is encoded by the *WRKY10* gene, and the preferential binding site of WRKY factors is TTGACC (A/T) or a W-box [12], [20]. There are three putative W-boxes in the *MINI3* promoter: W_1_-Box TTGACCA from nucleotides −257 to −262, W_3_-Box TTGACAA from nucleotides −1332 to −1337, and W_t_-Box TTGACAT from nucleotides −19 to −24 (Figure 5A). The *IKU2* promoter contains only one W-box, W_i_-Box TTGACTT from nucleotides −386 to −391 (Figure 5A). To examine whether MINI3 binds to the W-boxes, we examined the affinity of a recombinant MINI3 protein to four genomic DNA fragments that contained the corresponding W-boxes using an electrophoretic mobility shift assay (EMSA).

MINI3 bound directly to the W_1_-box in the *MINI3* promoter and the W_i_-box in the *IKU2* promoter (Figure 5C and 5D) but had a much weaker affinity with the W_3_-box in the *MINI3* promoter (Figure S2C). To confirm that MINI3 indeed bound W_1_-box or W_i_-box, we mutated the second T in the W-boxes to A and performed EMSA competition analyses (Figure 5B). The EMSA fragments that contained the mutated W_1_-box or W_i_-box failed to compete effectively with radio-labeled cognate wild type fragments (Figure 5C and 5D). Intriguingly, cold EMSA fragments that contained either wild type or mutated W_3_-box competed well with the radio-labeled cognate wild type fragment (Figure S2C). Either the binding of MINI3 to the W_3_-box was not specific or the second T in the W_3_-box is not essential for binding with MINI3. The binding of MINI3 to W_t_-box was clearly nonspecific because excess wild type cold W_t_-box failed to compete with radio-labeled W_t_-box (Figure S2D).

### SHB1 interacts with MINI3

**Figure 6:**
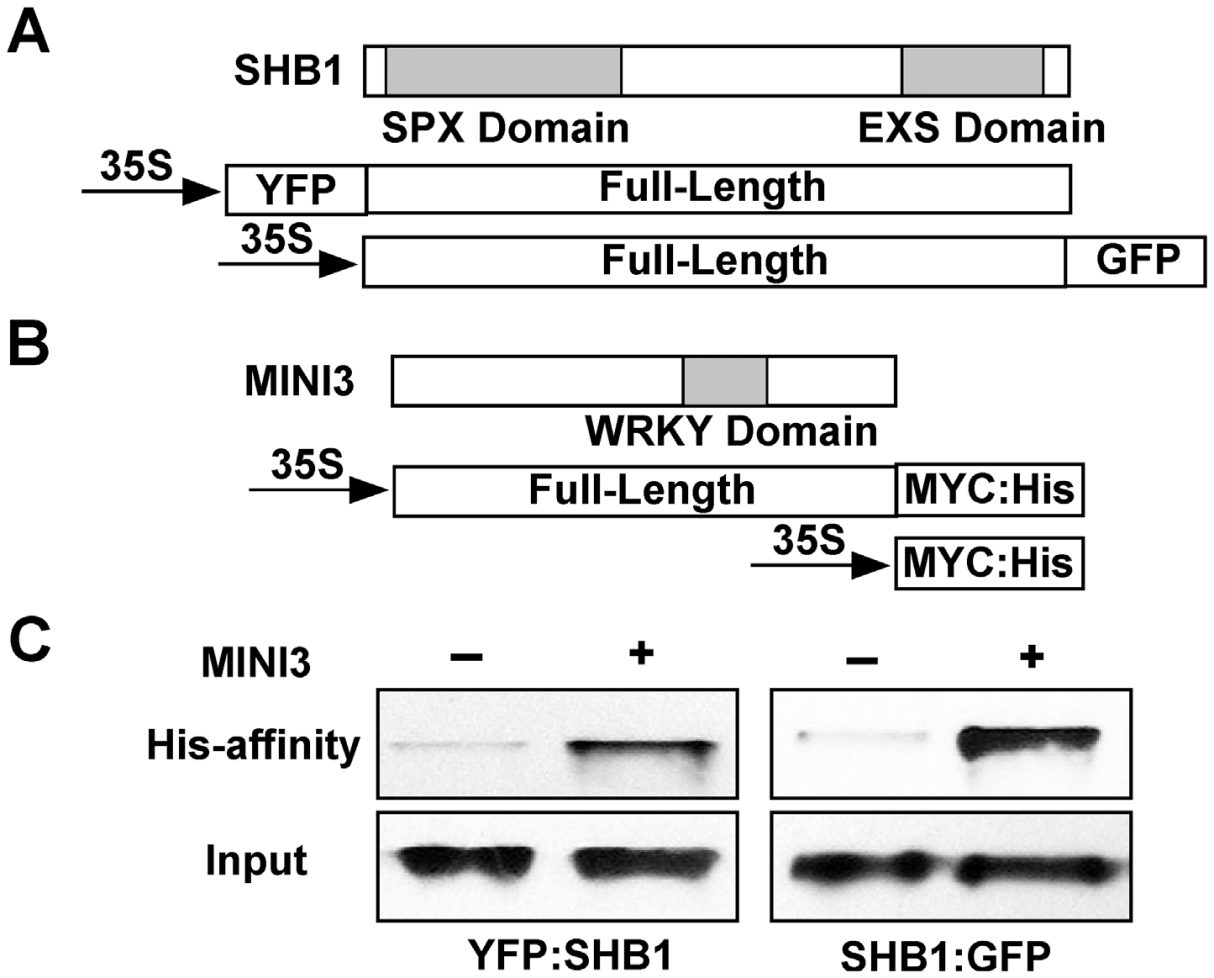
SHB1 interacts with MINI3. (A) A schematic drawing of full-length SHB1 fused to YFP at its N-terminus or GFP at its C-terminus under the control of the CaMV 35S promoter. The SPX and EXS domains of SHB1 are shaded in gray. (B) A schematic drawing of full-length MINI3 fused to MYC:His at its C-terminus under the control of the CaMV 35S promoter. The WRKY domain of MINI3 is shaded in gray. (C) His affinity-precipitation of YFP:SHB1 using MYC:His or MINI3:MYC:His from protein extracts prepared from Arabidopsis siliques (left), and of SHB1:GFP using MYC:His or MINI3:MYC:His from protein extracts prepared from *Nicotiana* leaves (right).

Both SHB1 and MINI3 associated with the same fragments in both *MINI3* and *IKU2* promoters, and the association of SHB1 with both promoters required MINI3 (Figure 3 and Figure 4). SHB1 and MINI3 may interact physically, and we performed an in vivo affinity-precipitation of MINI3:MYC:His for YFP:SHB1 in protein extracts prepared from *Arabidopsis* siliques (Figure 6A and 6B). MINI3:MYC:His did precipitate YFP:SHB1 but not MYC:His (Figure 6C, left). We then performed a parallel affinity-precipitation in a *Nicotiana* transient expression system. MINI3:MYC:His was also capable of precipitating SHB1:GFP in this system (Figure 6C, right).

### SHB1 N-terminus interacts with MINI3 N-terminus

**Figure 7:**
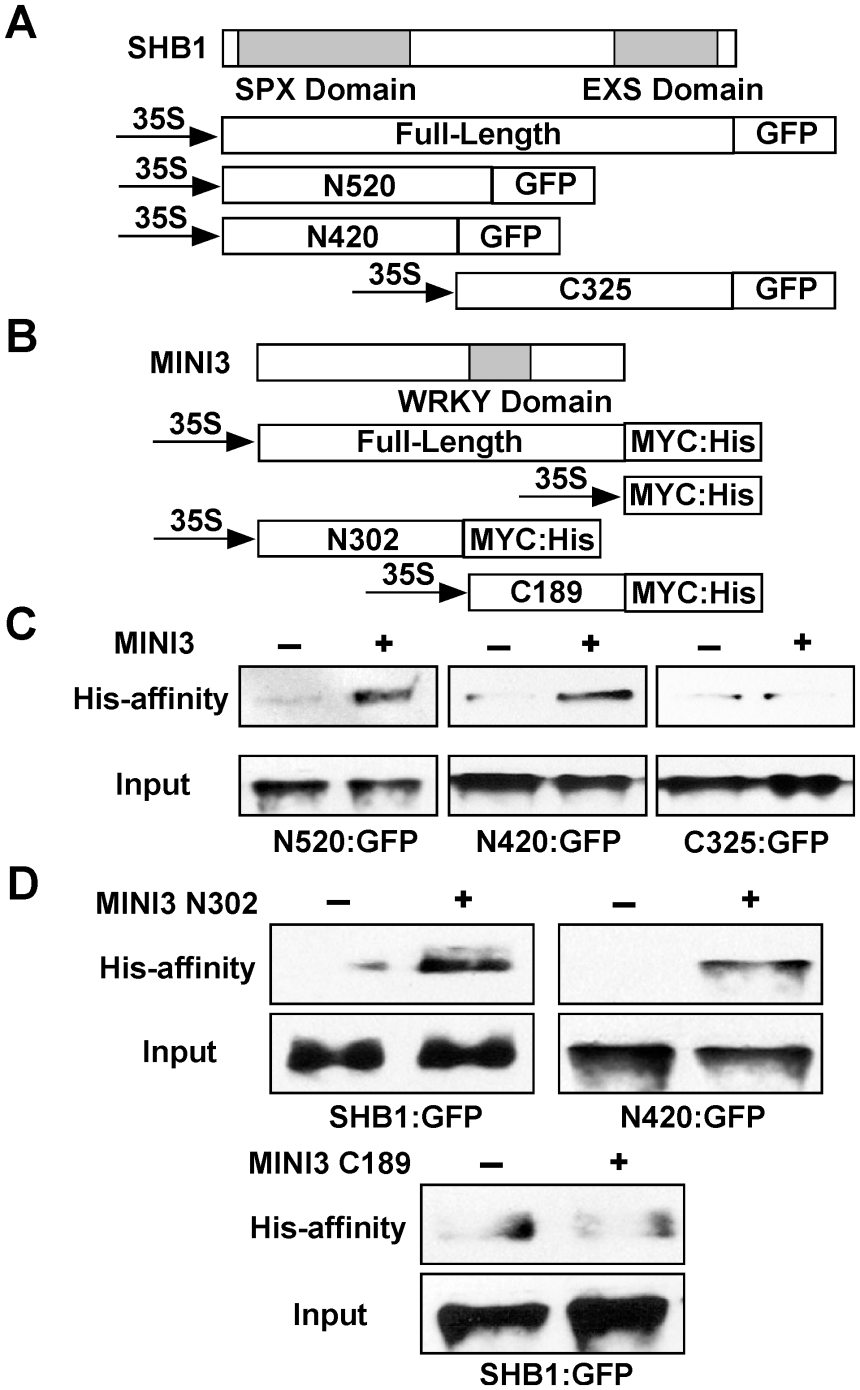
The SHB1 N-terminus interacts with the MINI3 N-terminus. (A) A schematic drawing of full-length and truncated SHB1 fused to GFP at its C-terminus under the control of the CaMV 35S promoter. The SPX and EXS domains of SHB1 are shaded in gray. (B) A schematic drawing of full-length and truncated MINI3 fused to MYC:His at its C-terminus under the control of the CaMV 35S promoter. The WRKY domain of MINI3 is shaded in gray. (C) His affinity-precipitation of SHB1 N520:GFP (left), N420:GFP (middle), or C325:GFP (right) using MYC:His or MINI3:MYC:His from protein extracts prepared from *Nicotiana* leaves. (D) His affinity-precipitation of SHB1:GFP (top left) or N420:GFP (top right) using MYC:His or MINI3 N302:MYC:His from protein extracts prepared from *Nicotiana* leaves. His affinity-precipitation of SHB1:GFP (bottom) using MYC:His or MINI3 C189:MYC:His from protein extracts prepared from *Nicotiana* leaves.

The most likely function of the SPX domain in SHB1 is to mediate protein-protein interactions [19]. The EXS domain contains several predicted trans-membrane helices, suggesting a membrane localization of the proteins [19], [21], [22]. However, SHB1 localizes to the nucleus, and the EXS domain in SHB1 may have a function distinct from those in other SYG1–like proteins. To map the interaction domain of SHB1 with MINI3, we performed an affinity precipitation of three truncated SHB1:GFP fusions, N520:GFP, N420:GFP, and C325:GFP, with MINI3:MYC:His in the *Nicotiana* transient expression system (Figure 7A and 7B). The N520:GFP and N420:GFP fusion proteins contain the N-terminal 520 or 420 amino acids, respectively, and include the putative SPX domain. The C325:GFP fusion protein contains the C-terminal 325 amino acids including the EXS domain (Figure 7A). MINI3:MYC:His precipitated both N520:GFP and N420:GFP (Figure 7C, left and middle) but not C325:GFP (Figure 7C, right). We conclude that SHB1 N-terminus mediates its interaction with MINI3, and its SPX domain may play an important role in this interaction.

The MINI3 protein contains 485 amino acids and belongs to the WRKY family of plant transcription factors [23]. MINI3 contains a conserved WRKY DNA binding domain between amino acids 306 and 365 and the remaining amino acid sequence does not reveal any significant sequence homologies to others in the database. We made an N-terminal MINI3 construct that contains the first 302 amino acids (N302:MYC:His) and a C-terminal MINI3 construct that contains the C-terminal 189 amino acids including the WRKY domain (C189:MYC:His; Figure 7B). The N302:MYC:His precipitated SHB1:GFP and the N420:GFP fusion protein (Figure 7D, top left and right). In contrast, the C189:MYC:His protein failed to precipitate SHB1:GFP (Figure 7D, bottom). The N-terminus of MINI3 but not its WRKY DNA binding domain is important for its interaction with SHB1.

### SHB1 activates expression from *MINI3* or *IKU2* promoters in a W-box-dependent manner

**Figure 8:**
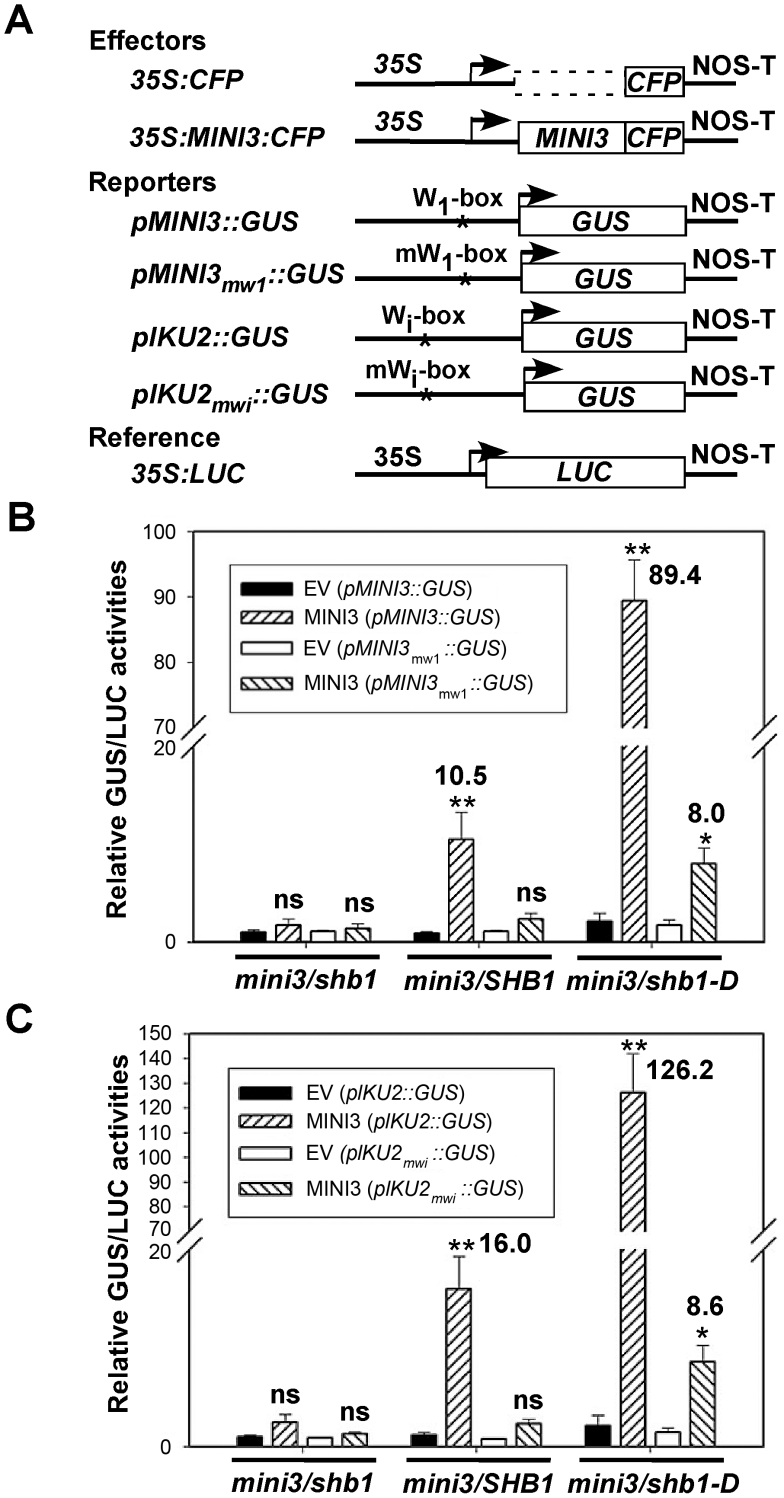
SHB1 activates transcription from *MINI3* and *IKU2* promoters. (A) A diagram showing the effector, reporter, and reference constructs used in the transient trans-activation assays. Full-length *MINI3* fused to CFP was used as effector and was driven by the CaMV 35S promoter, and an empty vector (EV) was used as a control. For reporters, the *GUS* gene was driven by either the wild-type *MINI3* or *IKU2* promoter or mutated W_1_-box in the *MINI3* promoter or mutated W_i_-box in the *IKU2* promoter and used as a reporter. Arrowheads indicate transcription start sites, and NOS-T represents polyadenylation signal from the nopaline synthase gene. The asterisk indicates the location of W-boxes in the *MINI3* or *IKU2* promoters. *LUC* gene driven by the CaMV 35S promoter was used as an internal reference. (B) *GUS* expression from the *MINI3* promoter with a wild-type (*pMINI3*::*GUS*) or a mutated (*pMINI3_m1_*::*GUS*) W_1_-box in the presence of an empty vector (EV) or MINI3 in *mini3*/*shb1*, *mini3*/*SHB1* or *mini3*/*shb1-D* backgrounds. (C) *GUS* expression from the *IKU2* promoter with a wild-type (*pIKU2*::*GUS*) or a mutated (*pIKU2_mwi_*::*GUS*) W_i_-box in the presence of an EV or MINI3 in *mini3*/*shb1*, *mini3*/*SHB1* or *mini3*/*shb1-D* bckgrounds. GUS activities were expressed in pico-moles per min per mg protein relative to LUC activities, which were expressed in light units per mg protein. The relative GUS/LUC activities for the EV and *pMINI3*::GUS pair (24 pico-moles/min) in B or the EV and *pIKU2*::GUS pair (52 pico-moles/min) in C were set to 1, and the remaining values were expressed as fold trans-activation. Data are calculated from at least three independent experiments as the mean plus or minus standard error (n≥3). The levels of significance of differences were determined using Student's *t*-test. ** *P*<0.01, * *P*<0.05, and ns, not significantly different.

MINI3 does not contain a recognizable trans-activation motif, and MINI3 may not activate the expression of *MINI3* or *IKU2* alone. We conducted in vivo trans-activation experiments in *Arabidopsis* siliques by bombarding 35S CaMV::MINI3 effectors together with *MINI3-* or *IKU2* promoter-GUS reporters into *mini3*/*shb1*, *mini3*/*SHB1*, or *mini3*/*shb1-D* developing siliques (Figure 8A). A CaMV 35S::LUC vector was also co-transformed to control for differences in transformation efficiency. When delivered back to *mini3*/*shb1* double mutant, MINI3 alone activated very little transcription from either the *MINI3* or the *IKU2* promoter over that of the empty vector (EV) control (Figure 8B and 8C). In contrast, SHB1 activated expression from both the *MINI3* and the *IKU2* promoters by 11- and 16-fold, respectively, when MINI3 was delivered to the *mini3*/*SHB1* background. Transcription from either the *MINI3* or the *IKU2* promoter was further activated up to 89- and 126-fold, respectively, in *mini3*/*shb1-D*.

When the high-affinity W_1_-box binding site in the *MINI3* promoter was mutated, the activation of expression from the *MINI3* promoter was almost undetectable in *mini3*/*SHB1* plants and decreased to 8.0-fold in *mini3*/*shb1*-*D* plants (Figure 8B). When the MINI3 Wi-box binding site in the *IKU2* promoter was mutated, the activation of expression from the *IKU2* promoter by SHB1 was similarly undetectable in *mini3*/*SHB1* plants and was reduced 8.6-fold in *mini3*/*shb1*-*D* plants (Figure 8C). A mutation in the low-affinity W_3_-box binding site slightly reduced the activation of *MINI3* expression by SHB1 to 6.3-fold in *mini3*/*SHB1* plants and 47.2-fold in *mini3*/*shb1*-*D* plants (Figure S3A and S3B). A mutation in the non-specific W_t_-box binding site had little affect on the activation by SHB1 (Figure S3A and S3C). Thus, the W_1_-box is the major binding site for MINI3, and the W_3_-box may play a subsidiary role to stabilize the DNA-protein interaction. In addition, the flanking sequences upstream or downstream of the core W-box may also exert secondary effects on the affinity of MINI3 to each particular W-box [23].

**Figure 9:**
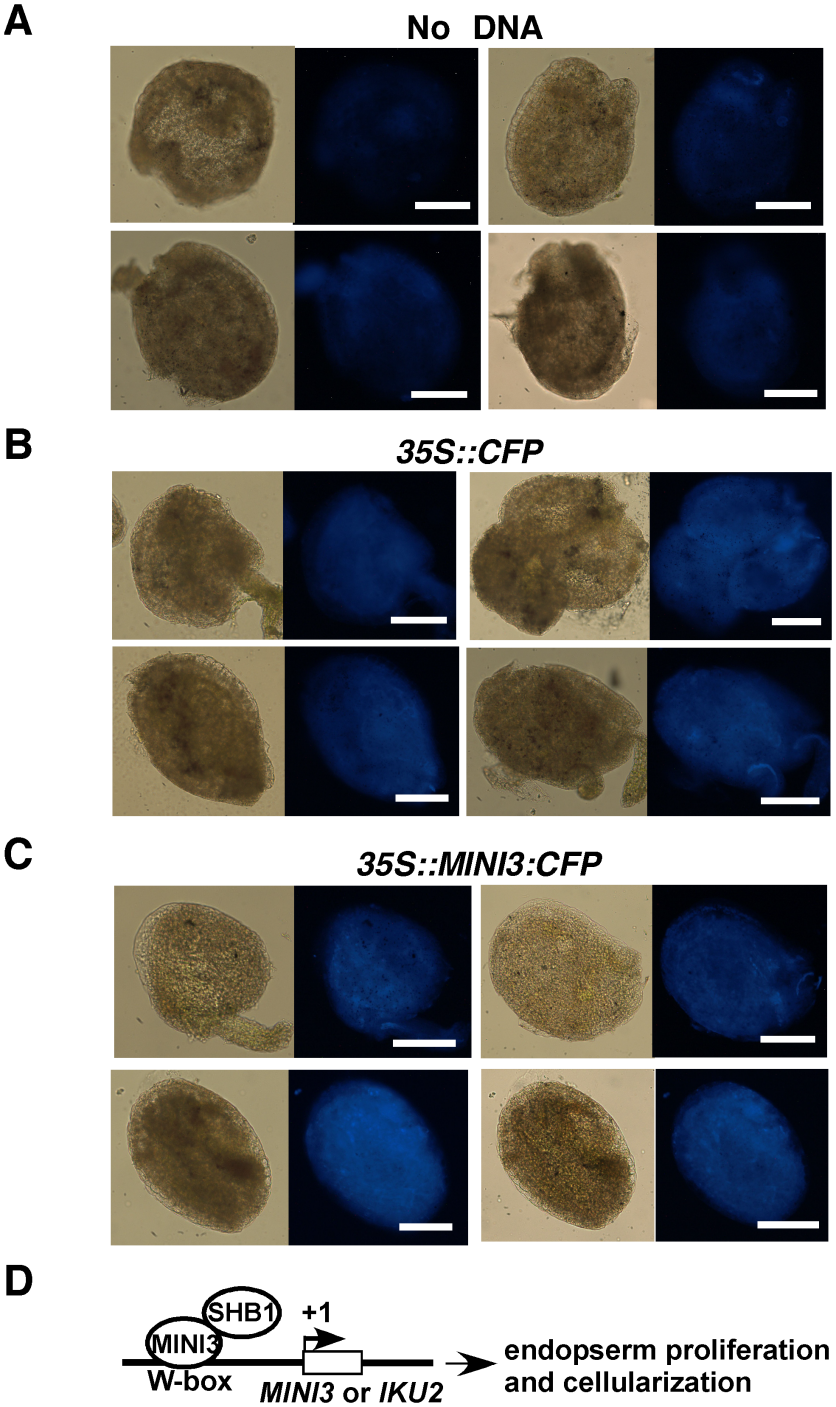
The CaMV 35S promoter drives expression of CFP or MINI3:CFP in transient bombardment experiments. CFP signals in seeds bombarded without DNA (A) and with 35::CFP (B) or 35S::MINI3:CFP (C) construct. (D) A model depicting the function of SHB1 in *MINI3* and *IKU2* expression. SHB1 is likely recruited to the *MINI3* and *IKU2* promoters by MINI3, which specifically recognizes particular W-box sequences in these promoters. SHB1 or SHB1 together with other proteins activates expression from the *MINI3*, *IKU2*, and/or other target gene promoters required for endosperm proliferation and seed cavity enlargement.

In the absence of MINI3, SHB1 induced little activation of expression from the promoters of *MINI3* and *IKU2* because SHB1 failed to target to these promoters (Figure S4). There was no notable activation of *pMINI3::GUS* or *pIKU2::GUS* expression in *MINI3*/*shb1* plants, and MINI3 alone was unable to activate transcription from either promoter (Figure S5). We did not observe an inhibitory effect of MINI3 on its own expression in this transient system as has been shown by others in stable transgenic plants [12]. To determine whether the 35S promoter drives the expression of CFP or MINI3:CFP in this transient system, we delivered no DNA, 35S::CFP vector, or 35S::MINI3:CFP vector to Ws seeds at various DAP but primarily at 3 to 4 DAP (Figure 9A to 9C). Figure 9A shows background signals in various seed tissues in the control (no DNA vector delivered). CFP signals were robust in tissues bombarded with either 35S::CFP or 35S::MINI3:CFP vector (Figure 9B and 9C).

## Discussion

### SHB1 activates the expression of *MINI3* and *IKU2* to trigger endosperm proliferation

In most species, endosperm in the syncytial phase undergoes cell expansion and proliferation to generate a large multinucleate cell close to the size of the mature seed [4], [5]. At 4 DAP, cellularization then partitions this syncytium into individual cells and marks the end of the maximum rate of endosperm proliferation [6]. Mutations in *MINI3* and *IKU2* cause premature cellularization of the endosperm and attenuate further proliferation of the cellularized endosperm [6], [12]. In contrast, the timing of endosperm cellularization is delayed in *shb1-D* [15]. The expression of *MIN3* and *IKU2* falls within a relatively narrow window, and the expression pattern of *SHB1* overlaps with *MINI3* and *IKU2* in the endosperm [12], [15]. The expression of *MIN3* and *IKU2* is undetectable before fertilization (Figures 1 and 2), and one of the most compelling reasons is that MINI3 inhibits its own expression [12]. Consequently, the expression of *IKU2* is also repressed because MINI3 is required for the proper expression of *IKU2* [12], [15]. The expression of *MINI3* and *IKU2* peaks at 3 DAP, before the formation of a large seed cavity at 4 DAP, and declines or becomes repressed again after 4 DAP (Figure 1 and Figure 2). It is not known how MINI3 represses its own expression before fertilization and 4 DAP. The activation of *MINI3* and *IKU2* expression between 2 and 3 DAP may involve both the de-repression of *MINI3* expression and an increase in SHB1 activity. The trigger for these two events remains unknown.

### MINI3 recruits SHB1 as a transcription activator

In the absence of SHB1, MINI3 induces little activation of the expression of *MINI3* or *IKU2* and the expression of *MINI3* and *IKU2* is enhanced in *mini3 SHB1* or *mini3 shb1-D* plants (Figure 8). MINI3 may function as a scaffold protein, and its N-terminus interacts with the N-terminus of SHB1 (Figure 7). Together with other proteins, the C-terminus of SHB1 may be capable of trans-activation. We propose a model where SHB1 is recruited by MINI3, which specifically binds W-box sequences in the promoters of *MINI3* and *IKU2*, to activate the transcription from the *MINI3* and *IKU2* promoters and/or other target genes required for endosperm development (Figure 9D). IKU1 is a nuclear VQ motif protein that also interacts with the N-terminus of MINI3 via its N-terminal VQ motif [16]. *IKU1* is expressed in the early developing endosperm prior to cellularization but GFP signals in *PIKU1*:*GFP:IKU1* plants are also detected in the integument or seed coats [16].

### Amount of stable and transient expression driven by the CaMV 35S promoter in the endosperm

It has been reported previously that cauliflower mosaic virus 35S promoter was not active during embryogenesis prior to the torpedo stage and that it did not show any activity in the syncytial endosperm [3]. We performed a MINI3 ChIP analysis in 35S::MINI3:GFP transgenic plants. Although we observed slightly higher MINI3:GFP expression compared with the background fluorescence of non-transgenic Ws plants, we are not convinced and do not present these images. Instead, we present data from six independent transgenic lines that each shows a consistently increase in seed mass compared with non-transgenic Ws plants (Figure 3C). MINI3 is normally expressed in the endosperm and the embryo, and its expression in the endosperm is required to maintain *IKU2* expression for endosperm proliferation and seed cavity enlargement. The observed seed phenotype may be due to the low expression of MINI3:GFP driven by the CaMV 35S promoter in the endosperm. In addition, in our experiments, we harvested siliques at approximately 4 DAP or at the early heart stage, and the embryo constitutes only a small volume of the seed at this stage. The weak expression of MINI3:GFP in embryo only does not account for our positive ChIP results. Therefore, the 35S::MINI:GFP transgenic plants may produce sufficient levels of tagged MINI3 for the ChIP analysis.

We also used a MINI3:CFP construct driven by the 35S promoter as an effector in our transient trans-activation analysis that was transfected via particle bombardment (Figure 8). We delivered CFP and MINI3:CFP vectors driven by the 35S promoter to seeds at 3 to 4 DAP, and crobust CFP signals were detected in tissues bombarded with the 35S::CFP or 35S::MINI3:CFP vector (Figure 9B and 9C). The strength of the CaMV 35S promoter in the endosperm may be low in stable transgenic plants [3]. The low expression level in the endosperm conferred by the CaMV 35S promoter may be due to specific chromatin structures surrounding the transcription factor genes that act on this promoter. The mechanism for transient expression may be different. In the case of agrobacterium-mediated transient expression, only a small percentage of the DNA molecules integrate into the host chromosomes but the pieces of DNA that do not integrate remain transcriptionally competent [24]. In a bombardment experiment, DNA vectors are delivered into the integument cells, the endosperm nucleus, and the large cytosolic fraction of the endosperm at 4 DAP, which is when the endosperm begins to cellularize and that embryo is tiny and at the globular or early heart stage. The DNA vectors bombarded into the endosperm nucleus may not integrate into the host chromosomes but are transcriptionally competent. RNA polymerase and transcription factors that are translated in the cytosol could act on the DNA vectors delivered to the cytosolic fraction of the endosperm. A strong indication of this possibility is the transcription from the *IKU2::GUS* construct in our transient system because the *IKU2* promoter confers expression that is endosperm-specific (Figure 2 and Figure 8C).

### Redundant pathways regulate endosperm proliferation

After cellularization, further proliferation and expansion of the endosperm ceases, and the timing of endosperm cellularization may shape the final seed size. Many genes that influence seed size and the timing of endosperm cellularization fall into three major categories. These three redundant pathways ensure a robust seed set for success in reproduction. AP2 and some MADS-box transcription factors comprise the first category. *AP2* encodes a plant-specific transcription factor with an AP2 DNA binding motif of 68 amino acids [25]. In an *ap2* mutant, the cellularization process is delayed and prolonged resulting in a larger embryo sac [26]. Several MADS-box proteins regulate endosperm development maternally. FEM111/AGAMOUS-LIKE80 (AGL80) regulates central cell development and the subsequent endosperm cellularization [27]. An *agl61* mutant shows a similar central cell and endosperm phenotype to *fem111*/*agl80* [28]. AGL80 interacts with AGL61 and appears to recruit AGL61 to the nucleus [29]. AGL80 also interacts with AGL62, and the endosperm undergoes early cellularization in an *agl62* mutant [18].

The second category includes five polycomb (PcG) proteins to form repressive FERTILIZATION INDEPENDENT SEED (FIS) complexes via histone methylation, and the imprinting of the polycomb genes by METHYL TRANSFERASE1 (MET1) and DECREASE IN DNA METHYLATION 1 (DDM1) [30]. In *fis* mutants, the female gametophytes initiate endosperm hyperproliferation without fertilization but the endosperm fails to cellularize. The paternal imprinting of *MEDEA (MEA* or *FIS1)* and *FIS2* is mediated by MET1, and loss of MET1 activity in Arabidopsis causes genome wide DNA hypomethylation at CpG dinucleotides [31], [32]. The *met1* mutation in the maternal genome causes larger seeds and results in a delay in endosperm cellularization and a larger endosperm volume [9]. In contrast, the *met1* mutation in the paternal genome produces smaller seeds due to early cellularization of the endosperm. A similar result was also observed in a reciprocal cross between wild type and *ddm1*, which carries a mutation in a gene required for genomic cytosine methylation [9].

SHB1 co-activator, a WRKY transcriptional factor, and a receptor protein kinase belong to the third category. In this report, we reveal the molecular actions and interactions of the key players but their connections to other pathways remain elusive. In addition, how the three pathways interact and integrate remains largely unknown. Previously, an interesting story had emerged that *AGL62* could mediate FIS protein function [18]. *AGL62* is strongly expressed in the endosperm during the syncytial phase, and its expression declines sharply before cellularization. In some *fis* mutants, the endosperm fails to cellularize and *AGL62* expression fails to decline [18]. The misexpression of *AGL62* in *fis* mutants may account for the cellularization phenotype of the *fis* mutants. Furthermore, whether the three pathways target the same set or different sets of genes required for endosperm development awaits further investigation.

## Materials and Methods

### Plant materials and mutant genotyping

*Arabidopsis* thaliana ecotype Wassilewskija (Ws) and Columbia-0 (Col) were used as wild type plants. The mutant lines *shb1-D*, *shb1* (SALK_128406), *mini3-2* (SALK_050364), and the *mini3-2 shb1-D* double mutant were described previously [15], [17]. The *shb1 mini3-2* double mutant was generated by crossing *shb1* to *mini3-2* and PCR-genotyped using the primers described previously [15]. Plants were grown under continuous light in a growth chamber at 21°C.

### Expression of *MINI3* and *IKU2*

The entire promoter regions of *MINI3* from −1398 to −4 and of *IKU2* from −806 to −1 were PCR-amplified from *Arabidopsis* genomic DNA. The stop codons of the adjacent genes are a few bp further upstream, and the regions used likely contain the majority of the regulatory information. The primer pairs used are TCAGGAGGTGACATCGAA and GACAAATCCTTAGGATGTC for *MINI3*, and TACGTACGTGTTGGTGGT and TGTTCTCTACGTCGGAAGGA for *IKU2*. The PCR fragments were cloned into the pCR8/GW/TOPO entry vector, and then recombined into the pMDC107 vector [33]. The constructs were transformed into *Arabidopsis* Ws or Col backgrounds by vacuum infiltration [34]. Four independent transgenic plants for each construct were further selected from multiple lines for a single T-DNA insertion based on their hygromycin resistance. The transgene in each of the four independent lines of either Ws or Col background was crossed into corresponding *shb1-D*, *shb1*, or *mini3* mutant backgrounds. Plants homozygous for the transgene were used for all analyses. Images were acquired using a Nikon C1si Laser Scanning Confocal Microscope (Nikon, Melville, NY) equipped with a three-channel PMT detector. The tissue sample was excited at 488 nm for GFP fluorescence.

### ChIP analysis

*MINI3* genomic DNA was PCR-amplified and cloned into the pCR8/GW/TOPO vector, and then recombined into the pMDC83 vector [33]. The primers used were CGCGGATCCGATGAGTGATTTTGATC and CGCGGATCCCATGTCGACACCAAACT. This 35S:MINI3:GFP construct was transformed into *Arabidopsis* by vacuum infiltration [34]. The ChIP-PCR experiment was described previously, and chromatin DNA was sonicated and sheared to approximately 0.3 to 2 kb [15], [35]. The seed mass of several Ws and independent 35S::MINI3:GFP transgenic lines was measured as described previously [15].

### Electrophoretic Mobility Shift Assay (EMSA)

Full-length *MINI3* cDNA was cloned into the pDEST17 vector with a His-tag at its C-terminus. Total soluble protein was prepared from E. *coli* in binding buffer that contained 25 mM Hepes-KOH pH 7.2, 40 mM KCl, 1 mM DTT, 1 mM EDTA, 10% glycerol, and a complete set of protease inhibitors. The recombinant proteins were purified as pdescribed previously, and were dialyzed against the binding buffer [36]. An empty pDEST17 vector was used as a negative control.

EMSA was performed as described previously [36]. Three DNA fragments from the *MINI3* promoter and one fragment from the *IKU2* promoter were approximately 150 bp in length and were PCR-labeled with alpha-^35^S dATP. The Primer pairs used were GAGGATATAGTGGGTCT and GACAAATCCTTAGGATGTC for fragment containing the W_1_-box, TCAGGAGGTGACATCGAA and GACATTACTGTCATCGT for fragment containing the W_3_-box, ATTTGCTGCCACTCTTTGA and GCTGCTAGCGTTTTCTGA for fragment containing the W_t_-box, and TACGTACGTGTTGGTGGT and CCAACGGATATGATTCA for fragment containing the W_i_-box. The binding reaction was initiated by mixing 2 µg poly-dIdC, 3 ng radiolabeled probe (300 cpm/ng), 0.01 µM ZnCl_2_, various amounts of cold competitor, and 1 µg purified protein in 20 µl binding buffer. The reaction was incubated at room temperature for 30 min and separated on a 6% polyacrylamide gel in 0.25× TBE buffer. All W-boxes were also mutated by changing the second T to A using the QuikChange Site-Directed Mutagenesis kit (Stratagene, La Jolla, CA).

### *In vivo* affinity-precipitation

*SHB1* genomic DNA was PCR-amplified and cloned into the pCR8/GW/TOPO vector, and then recombined into the pEARLEYGATE104 (35S:YFP:SHB1) [37]. The primers used were CATGCCATGGTGAGGTTTGGGAAAGA and ATTGTTATGATGATCTCCA. *MINI3* genomic DNA in the pCR8/GW/TOPO vector, as described in the Methods for the ChIP assay, was recombined into a modified pCAMBIA1390 vector, C-terminal to a MYC:His tag [38]. All constructs were introduced to *Arabidopsis* using the *Agrobacterium*-mediated vacuum infiltration method [34]. Either the 35S:MYC:His or the 35S:MINI3:MYC:His transgene was crossed into the 35S:YFP:SHB1 background. Approximately 4 g of developing siliques at 3 to 4 DAP were harvested, frozen in liquid nitrogen, and ground in 8 ml of cold co-precipitation buffer (50 mM Tris-Cl, pH 7.5, 150 mM NaCl, 10% glycerol, 0.5% NP-40, 10 mM imidazole, and a full set of proteinase inhibitors). After centrifuging at 30,000 *g* and 4°C for 30 minutes, the supernatant was incubated with 150 µl pre-equilibrated nickel-agarose beads (Qiagen, Valencia, CA) at 4°C for 2 hr [38]. The incubation mixture was washed 5 times using cold co-precipitation buffer, and the proteins attached to the column were eluted using 100 µl cold co-precipitation buffer containing 200 mM imidazole. Approximately 48 µl of the elution was mixed with 12 µl 5x SDS loading buffer and loaded into a 10% SDS-PAGE gel. After blotting onto a membrane, the blotted proteins were probed using an anti-GFP antibody or anti-MYC primary antibody (Santa Cruz Biotechnology, Santa Cruz, CA).

*In vivo* affinity-precipitation in a transient *Nicotiana* expression system was performed as described [39]. *MINI3* truncated derivatives, N302 and C189, were PCR-amplified and cloned into a pCR8/GW/TOPO vector and recombined into the modified pCAMBIA1390 vector as described above. Primer pairs used were CGCGGATCCGATGAGTGATTTTGATG and GTCGCTTTCCATCTGAAG for N302, and CTTCAGATGGAAAGCGAC and CATGTCGACACCAAACTT for C189. The SHB1 full-length and SHB1 truncated derivatives, N520:GFP, N420:GFP and C325:GFP, in the pEZT-NL vector have been described previously [40]. Approximately 10 g of *Nicotiana* leaves were harvested 5 days after the initial infiltration, frozen in liquid nitrogen, and ground in 10 ml of cold co-precipitation buffer [41].

### Transient trans-activation assay

The promoter regions of *MINI3* and *IKU2* in pCR8/GW/TOPO entry vectors were recombined into the pMDC163 vector as reporter constructs [33]. The mutations in each W-box were introduced as described for the MSA assay. *MINI3* genomic DNA in the pCR8/GW/TOPO vector, as described for the ChIP assay, was recombined into the pEARLAYGATE102 vector as an effector together with the empty vector as a control [37]. A 35S::LUC vector harboring firefly luciferase under the control of the cauliflower mosaic virus 35S promoter was co-bombarded as an internal standard.

Transient expression analysis in *Arabidopsis* siliques was performed as described previously [42]. Tungsten particles (Bio-Rad, Hercules, CA) were coated with 1 µg of the reporter plasmid, 1 µg of the effector plasmid, and 30 ng of internal control plasmid suspended in ethanol. *Arabidopsis* siliques at 3 to 4 DAP were harvested, placed in a 3-cm-diameter circle in the center of a 10-cm Petri plate with ½ MS and 0.8% (w/v) agar, and then incubated for 2 h in a growth chamber prior to bombardment. The tungsten particles were delivered using the PDS-1000/He Biolistic Particle Delivery System (Bio-Rad, Hercules, CA). After bombardment, the siliques were incubated for 20 h (12 h light/8 h dark) at 21°C in a growth chamber, harvested, and then frozen in liquid nitrogen. Plant extracts were prepared for GUS activity assay [41]. The LUC activity in the extracts was measured using a Luciferase Assay System (Promega, Madison, WI). GUS activity was normalized to LUC activity.

### Accession numbers

The sequences of the genes described in this report can be found in the *Arabidopsis* Genome Initiative database with the following accession numbers: *SHB1* (At4G25350), *MINI3* (At1G55600), *IKU2* (At3G19700), and *UBQ10* (At4G05320).

## Supporting Information

Figure S1 The SHB1 mutation shows a partial affects on the expression of *pMINI3::GFP*. Expression of *pMINI3::GFP* in Col wild type, *shb1*, and *mini3* seeds from 1 to 5 days after pollination (DAP). Images on the left (1 to 2 DAP) or at the top (3 to 5 DAP) are of bright field and images on the right (1 to 2 DAP) or at the bottom (3 to 5 DAP) are of GFP fluorescence. Scale bars, 100 µm.
(TIF)

Figure S2 MINI3 binds the W_3_-box weakly and the W_t_-box nonspecifically. (A) A schematic diagram of the *MINI3* loci, the three amplicons (M1, M2, and M3) used for the ChIP-qPCR analysis, and the position of the W-boxes in the *MINI3* promoter. Rectangles represent genes and numbers indicate genomic nucleotide sequence coordination. The arrowhead indicates the transcription start site, and the asterisk indicates the W-boxes likely recognized by MINI3. (B) The nucleotide sequences of W_3_-box, mutated W_3_-box (mW_3_-box), W_t_-box, and mutated W_t_-box (mW_t_-box). Core sequences are shaded in black, and mutated nucleotides are shaded in gray. (C and D) EMSA analysis of the binding of MINI3 to W_3_-box (C) or W_t_-box (D) in the *MINI3* promoter. Cold wild type or mutated W_3_-box and W_t_-box competitors were used at a molar excess of 5X, 10X, or 50X.
(TIF)

Figure S3 A mutation in the W_3_-box, but not the W_t_-box, shows a partial affect on transient expression from the *MINI3* or *IKU2* promoters. (A) A diagram showing the effector, reporter, and reference constructs used in the transient trans-activation assays. Full-length *MINI3* fused to CFP was driven by the CaMV 35S promoter as an effector, and an empty vector (EV) was used as a control. *GUS* gene was driven by either wild type or a mutated W_3_-box or W_t_-box in the *MINI3* promoter and used as a reporter. Arrowheads indicate transcription start sites, and NOS-T represents polyadenylation signal from the nopaline synthase gene. The asterisk indicates the location of the W-boxes in the *MINI3* promoter. The *LUC* gene driven by the CaMV 35S promoter was used as an internal reference. (B) *GUS* expression from the *MINI3* promoter with a wild-type (*pMINI3*::*GUS*) or a mutated (*pMINI3_mw3_*::*GUS*) W_3_-box in the presence of an empty vector (EV) or MINI3 in *mini3*/*shb1*, *mini3*/*SHB1* or *mini3*/*shb1-D* backgrounds. (C) *GUS* expression from the *MINI3* promoter with a wild-type (*pMINI3*::*GUS*) or a mutated (*pMINI3_mwt_*::*GUS*) W_t_-box in the presence of an EV or MINI3 in *mini3*/*shb1*, *mini3*/*SHB1* and *mini3*/*shb1-D* backgrounds. GUS activities were expressed in pico-moles per min per mg protein relative to LUC activities, which were expressed in light units per mg protein. The relative GUS/LUC activity for the EV and *pMINI3*::GUS pair (24 pico-moles/min; shown in Figure 7B) was set to 1 and the remaining values were expressed as fold trans-activation. Data were calculated from at least three independent experiments as the mean plus or minus the standard error (n≥3). The levels of significance of differences were determined using the Student's *t*-test. ** *P*<0.01, * *P*<0.05, and ns, not significantly different.
(TIF)

Figure S4 SHB1 is not targeted to either the *MINI3* or *IKU2* promoter in the absence of MINI3. (A) *GUS* is expressed from a wild type *MINI3* promoter (*pMINI3*::*GUS*) in the absence of MINI3:CFP in *mini3*/*shb1*, *mini3*/*SHB1* and *mini3*/*shb1-D*. (B) *GUS* is expressed from a wild type *IKU2* promoter (*pIKU2*::*GUS*) in the absence of MINI3:CFP in *mini3*/*shb1*, *mini3*/*SHB1* and *mini3*/*shb1-D*. GUS activities were expressed in pico-moles per min per mg protein relative to LUC activities, which were expressed in light units per mg protein. The relative GUS/LUC activity in *mini3*/*shb1* was set to 1 and the remaining values are expressed as fold trans-activation. Data were calculated from at least three independent experiments as the mean plus or minus the standard error (n≥3). The levels of significance of differences were determined using the Student's *t*-test.
(TIF)

Figure S5 MINI3 does not activate the expression of *MINI3* or *IKU2* in the absence of SHB1. (A) *GUS* is expressed from a wild type *MINI3* promoter (*pMINI3*::*GUS*) in *mini3*/*shb1* and *MINI3*/*shb1*. (B) *GUS* is expressed from a wild type *IKU2* promoter (*pIKU2*::*GUS*) in *mini3*/*shb1* and *MINI3*/*shb1*. GUS activities were expressed in pico-moles per min per mg protein relative to LUC activities, which were expressed in light units per mg protein. The relative GUS/LUC activity in *mini3*/*shb1* was set to 1 and the remaining values were expressed as fold trans-activation. Data were calculated from at least three independent experiments as the mean plus or minus the standard error (n≥3). The levels of significance of differences were determined using the Student's *t*-test.
(TIF)
