# Supplementary figures and images for: A WRKY Transcription Factor Recruits the SYG1-Like Protein SHB1 to Activate Gene Expression and Seed Cavity Enlargement
Source: PLoS Genet. 2013 Mar 7;9(3):e1003347. doi: 10.1371/journal.pgen.1003347 (PMC3591269; doi:10.1371/journal.pgen.1003347)

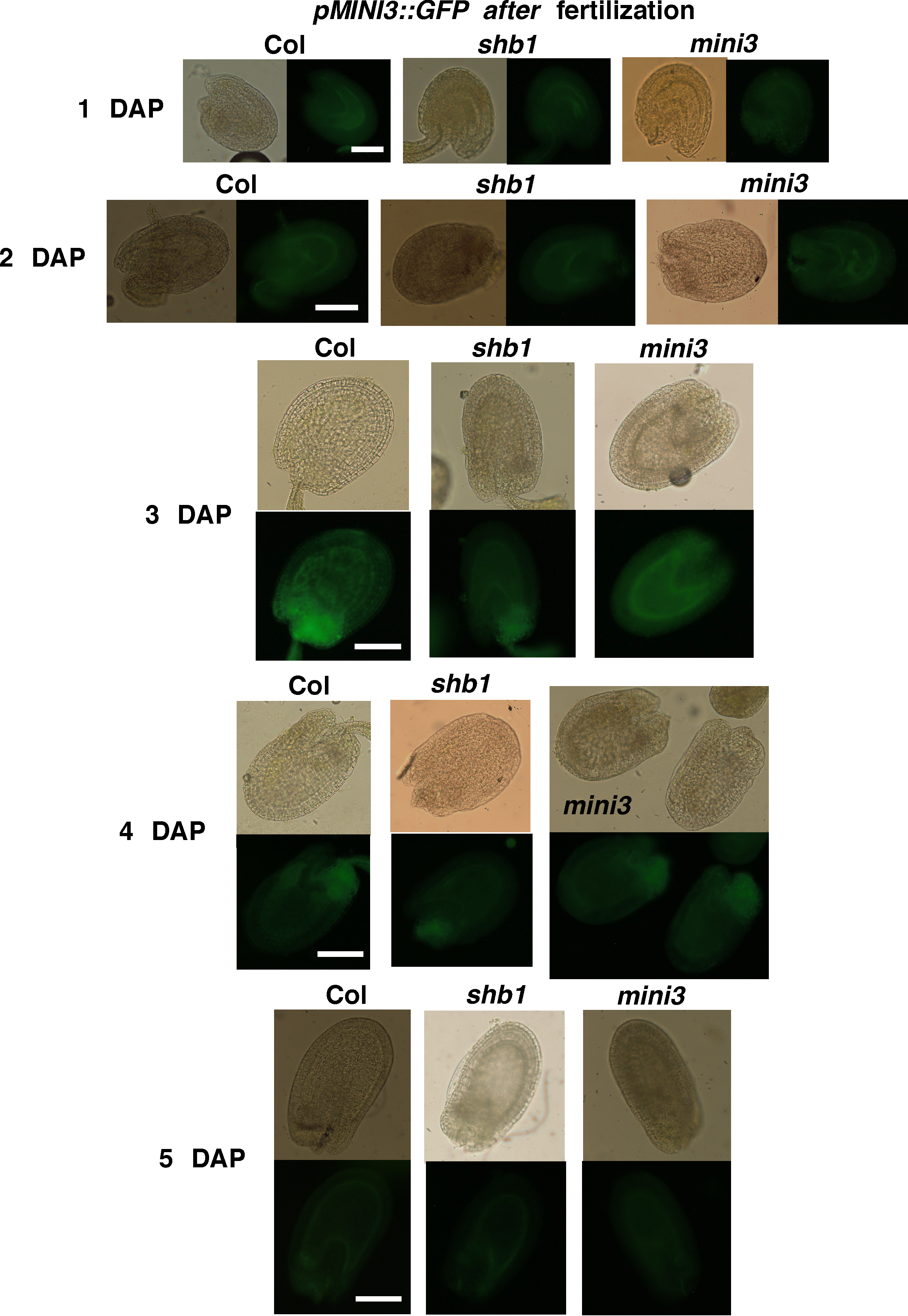

Supplement: Figure S1 — The SHB1 mutation shows a partial affects on the expression of pMINI3::GFP. Expression of pMINI3::GFP in Col wild type, shb1, and mini3 seeds from 1 to 5 days after pollination (DAP). Images on the left (1 to 2 DAP) or at the top (3 to 5 DAP) are of bright field and images on the right (1 to 2 DAP) or at the bottom (3 to 5 DAP) are of GFP fluorescence. Scale bars, 100 µm. (TIF) [file pgen.1003347.s001.tif]

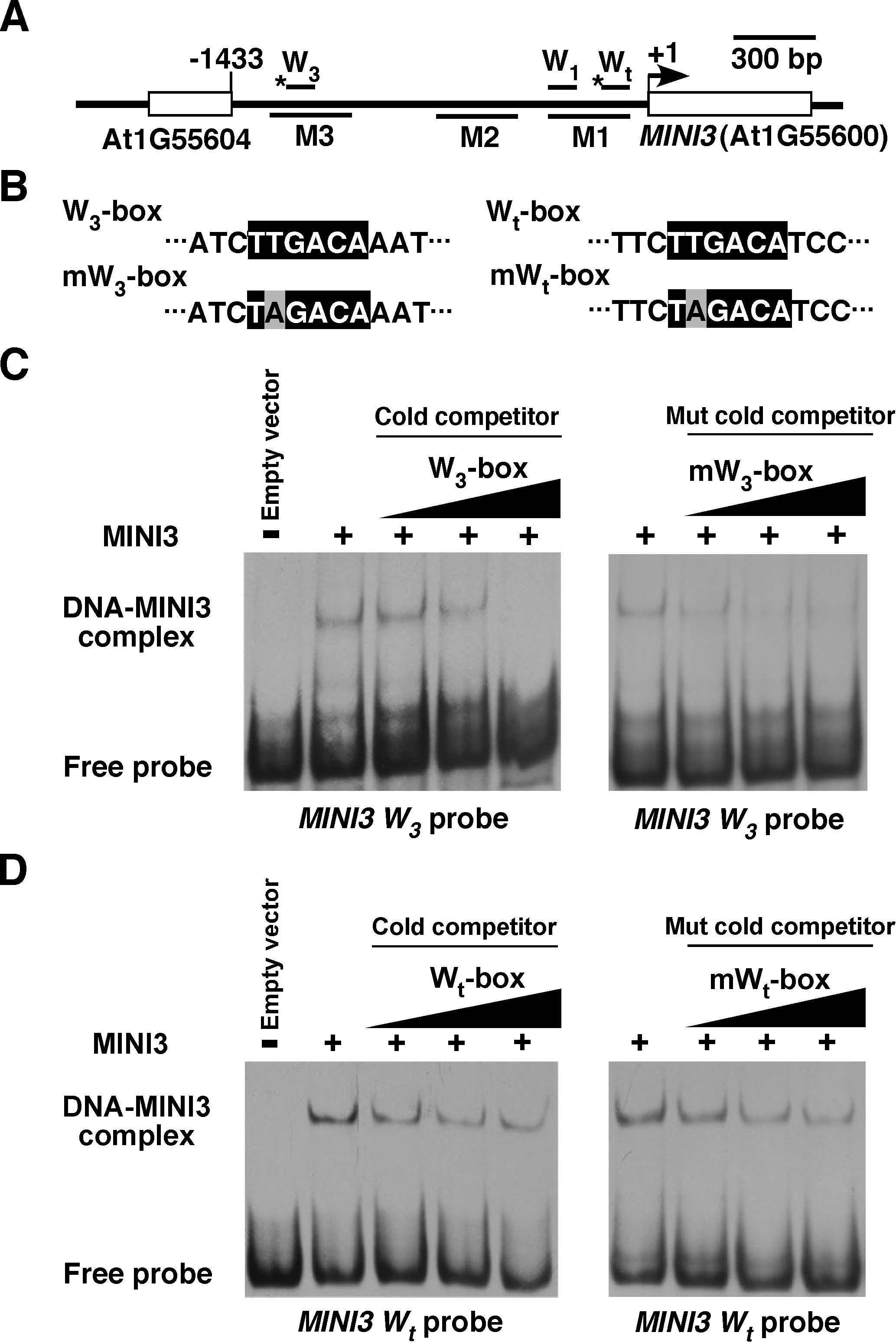

Supplement: Figure S2 — MINI3 binds the W3-box weakly and the Wt-box nonspecifically. (A) A schematic diagram of the MINI3 loci, the three amplicons (M1, M2, and M3) used for the ChIP-qPCR analysis, and the position of the W-boxes in the MINI3 promoter. Rectangles represent genes and numbers indicate genomic nucleotide sequence coordination. The arrowhead indicates the transcription start site, and the asterisk indicates the W-boxes likely recognized by MINI3. (B) The nucleotide sequences of W3-box, mutated W3-box (mW3-box), Wt-box, and mutated Wt-box (mWt-box). Core sequences are shaded in black, and mutated nucleotides are shaded in gray. (C and D) EMSA analysis of the binding of MINI3 to W3-box (C) or Wt-box (D) in the MINI3 promoter. Cold wild type or mutated W3-box and Wt-box competitors were used at a molar excess of 5X, 10X, or 50X. (TIF) [file pgen.1003347.s002.tif]

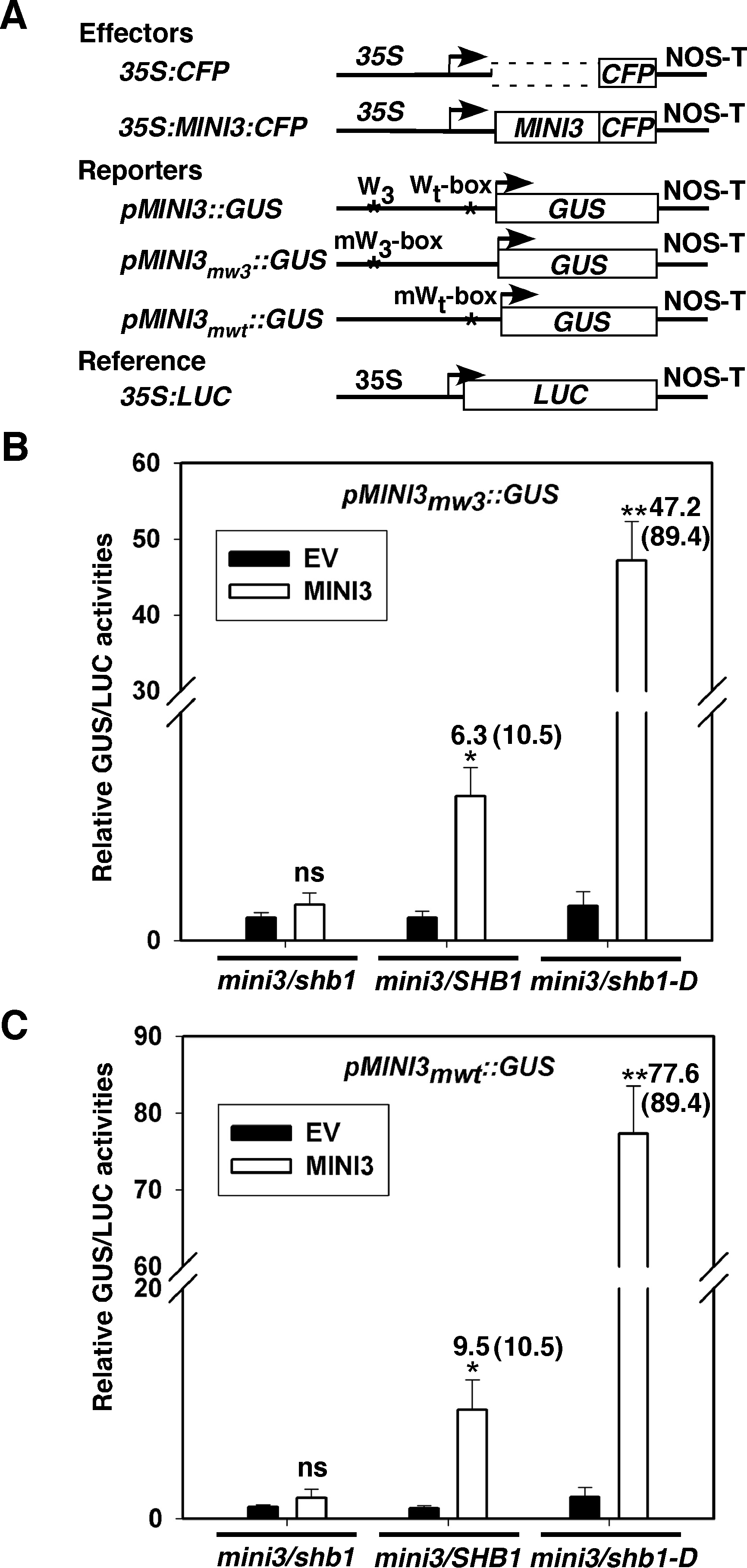

Supplement: Figure S3 — A mutation in the W3-box, but not the Wt-box, shows a partial affect on transient expression from the MINI3 or IKU2 promoters. (A) A diagram showing the effector, reporter, and reference constructs used in the transient trans-activation assays. Full-length MINI3 fused to CFP was driven by the CaMV 35S promoter as an effector, and an empty vector (EV) was used as a control. GUS gene was driven by either wild type or a mutated W3-box or Wt-box in the MINI3 promoter and used as a reporter. Arrowheads indicate transcription start sites, and NOS-T represents polyadenylation signal from the nopaline synthase gene. The asterisk indicates the location of the W-boxes in the MINI3 promoter. The LUC gene driven by the CaMV 35S promoter was used as an internal reference. (B) GUS expression from the MINI3 promoter with a wild-type (pMINI3::GUS) or a mutated (pMINI3mw3::GUS) W3-box in the presence of an empty vector (EV) or MINI3 in mini3/shb1, mini3/SHB1 or mini3/shb1-D backgrounds. (C) GUS expression from the MINI3 promoter with a wild-type (pMINI3::GUS) or a mutated (pMINI3mwt::GUS) Wt-box in the presence of an EV or MINI3 in mini3/shb1, mini3/SHB1 and mini3/shb1-D backgrounds. GUS activities were expressed in pico-moles per min per mg protein relative to LUC activities, which were expressed in light units per mg protein. The relative GUS/LUC activity for the EV and pMINI3::GUS pair (24 pico-moles/min; shown in Figure 7B) was set to 1 and the remaining values were expressed as fold trans-activation. Data were calculated from at least three independent experiments as the mean plus or minus the standard error (n≥3). The levels of significance of differences were determined using the Student's t-test. ** P<0.01, * P<0.05, and ns, not significantly different. (TIF) [file pgen.1003347.s003.tif]

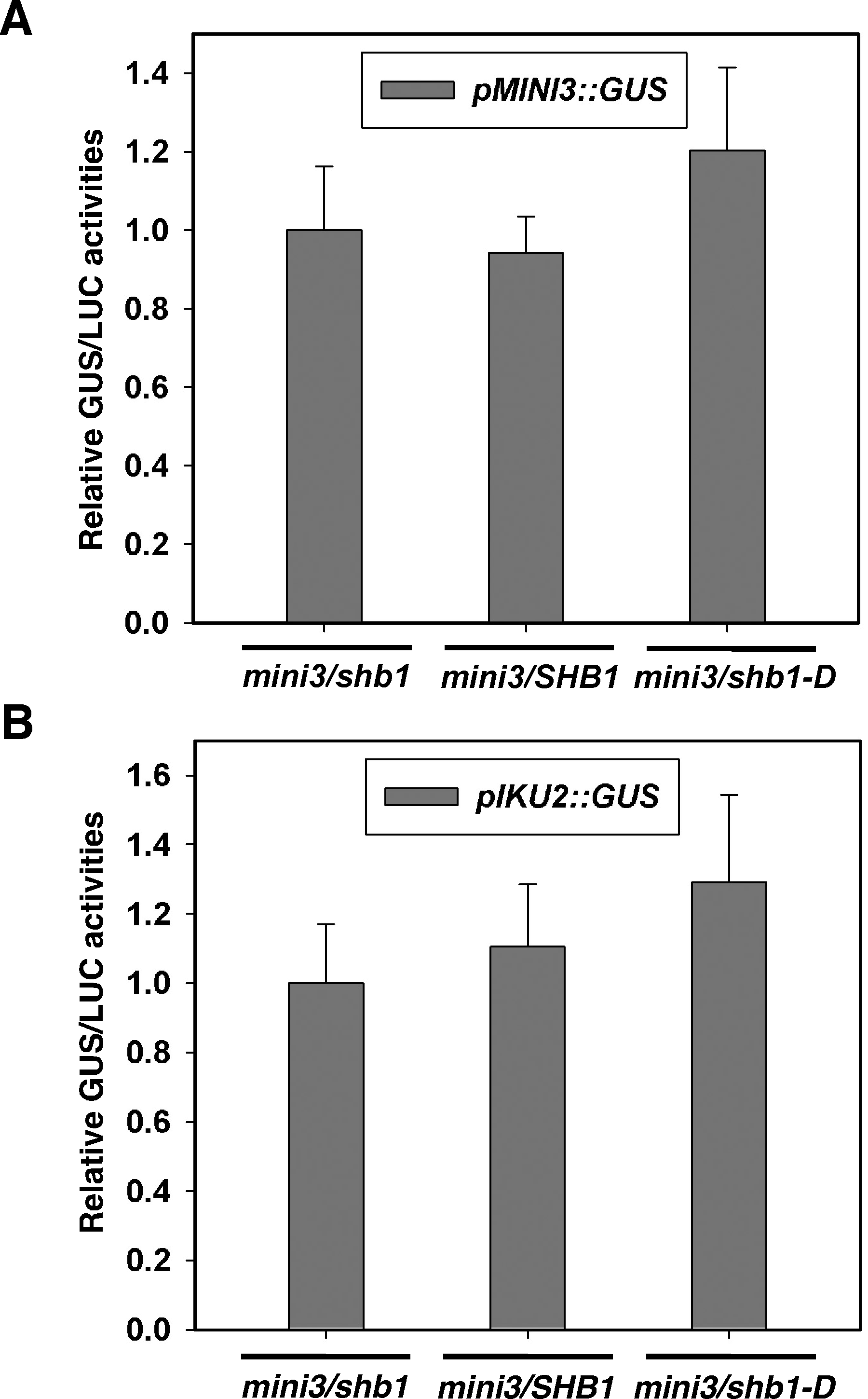

Supplement: Figure S4 — SHB1 is not targeted to either the MINI3 or IKU2 promoter in the absence of MINI3. (A) GUS is expressed from a wild type MINI3 promoter (pMINI3::GUS) in the absence of MINI3:CFP in mini3/shb1, mini3/SHB1 and mini3/shb1-D. (B) GUS is expressed from a wild type IKU2 promoter (pIKU2::GUS) in the absence of MINI3:CFP in mini3/shb1, mini3/SHB1 and mini3/shb1-D. GUS activities were expressed in pico-moles per min per mg protein relative to LUC activities, which were expressed in light units per mg protein. The relative GUS/LUC activity in mini3/shb1 was set to 1 and the remaining values are expressed as fold trans-activation. Data were calculated from at least three independent experiments as the mean plus or minus the standard error (n≥3). The levels of significance of differences were determined using the Student's t-test. (TIF) [file pgen.1003347.s004.tif]

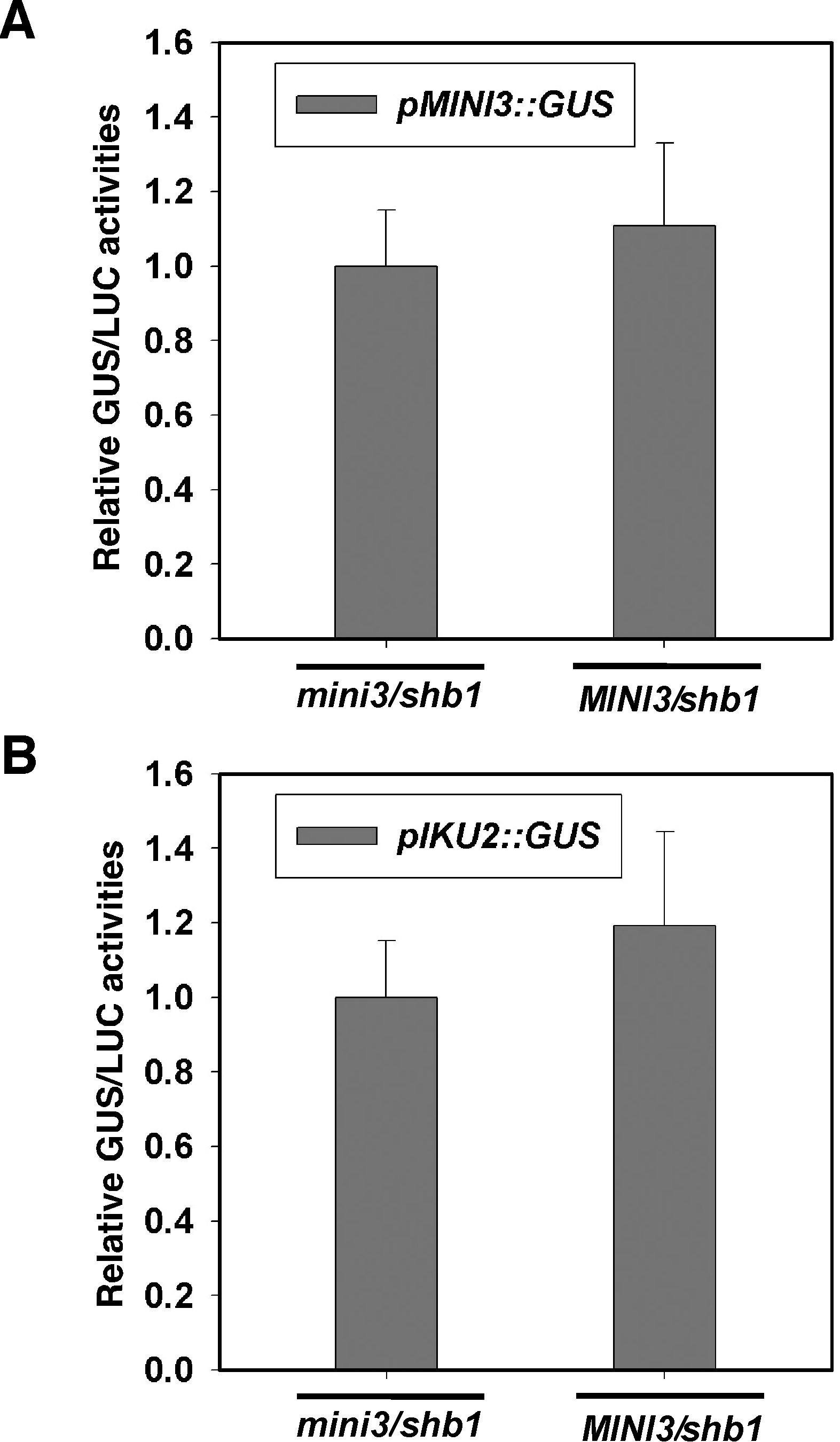

Supplement: Figure S5 — MINI3 does not activate the expression of MINI3 or IKU2 in the absence of SHB1. (A) GUS is expressed from a wild type MINI3 promoter (pMINI3::GUS) in mini3/shb1 and MINI3/shb1. (B) GUS is expressed from a wild type IKU2 promoter (pIKU2::GUS) in mini3/shb1 and MINI3/shb1. GUS activities were expressed in pico-moles per min per mg protein relative to LUC activities, which were expressed in light units per mg protein. The relative GUS/LUC activity in mini3/shb1 was set to 1 and the remaining values were expressed as fold trans-activation. Data were calculated from at least three independent experiments as the mean plus or minus the standard error (n≥3). The levels of significance of differences were determined using the Student's t-test. (TIF) [file pgen.1003347.s005.tif]
